# Dynamics of stand productivity in Moso bamboo forest after strip cutting

**DOI:** 10.3389/fpls.2022.1064232

**Published:** 2022-10-31

**Authors:** Yaxiong Zheng, Shaohui Fan, Xiao Zhou, Xuan Zhang, Fengying Guan

**Affiliations:** ^1^ International Center for Bamboo and Rattan, Key Laboratory of National Forestry and Grassland Administration, Beijing, China; ^2^ National Location Observation and Research Station of the Bamboo Forest Ecosystem in Yixing, National Forestry and Grassland Administration, Yixing, China

**Keywords:** Moso bamboo forest, strip clearcutting, stand characteristics, productivity, Correlation analysis

## Abstract

Strip cutting can effectively reduce the cutting cost of bamboo forests and promote the transformation and upgradation of bamboo forests through mechanization and modernization. Despite the rapid accumulation of Moso bamboo biomass, the dynamics of five years changes in stand characteristics and productivity after cutting remain unclear. This is critical for formulating efficient bamboo forest management measures. In this paper, plots with an 8 m width strip cut (SC) and respective reserved belts (RB) were selected as the research object, and the traditional management forest (CK) as control. The dynamic characteristics of stand, biomass distribution pattern, and productivity change in the different treatment plots were studied for 5 years after cutting. The results showed that cutting increased the number of shoots and new bamboo, and decreased the diameter at breast height, height to crown base, and height of new bamboo (*p*<0.05). Cutting reduces the productivity of both SC and RB, and allocates more biomass to the bamboo leaves to capture light in SC (*p*<0.05). Over time, the characteristics of new bamboo in SC reached the level of CK, and the density of standing bamboo, and productivity, were higher than those in CK. However, the number and productivity of new bamboo decreased significantly in the RB (*p*<0.05), which reflected the density restriction effect of bamboo forest. Further analysis showed that the increase in productivity in SC and CK was mainly from Moso bamboo at II and III “du”, which positively correlated with the soil contents of total nitrogen, total phosphorus, and available phosphorus. It was suggested that after three On-year restorations, the SC could reach the level of CK, however it is necessary to density manage RB from the second On-year after cutting.

## Introduction

Moso bamboo (*Phyllostachys edulis*) is an important forest resource in China and has important economic (Jiang, 2007) and ecologic value (Li et al., 2015). According to the ninth Forest Resources Inventory report, China has a total of 6.41 million hm^2^ of bamboo forests (Administration, 2019). Among them, there is 4.67 million hm^2^ of Moso bamboo forests, accounting for 72.96% of the country’s total bamboo forests. However, the shortage of labor resources has become the main factor restricting bamboo cultivation and industrial development (Zheng et al., 2021). Therefore, according to the physiological integration characteristics of Moso bamboo, bamboo forest management experts proposed a strip cutting method to reduce cutting costs (Fan et al., 2018). Studies on the dynamic characteristics of bamboo recovery using different cutting widths showed that the number of shoots per unit area increased significantly; the number of shoots and bamboo initially increased, and then decreased with increasing cutting intensity (Wang, 2021). Meanwhile, the quality of new bamboo deteriorated after cutting (Zeng et al., 2019). From the different experimental areas, the consistent conclusion drawn was that the structure of the new bamboo forest and soil environment is optimal and suitable for new bamboo development in plots harvested with 6–9 m bandwidth. This is the suitable width for bamboo forest strip cutting (Zeng, 2019; Zhan, 2019; Wu, 2020; Wang, 2021). The clonal integration of bamboo makes it possible to harvest the culms and shoots (Franklin, 2006).

For forest management and disturbance care, bamboo cutting has affected biodiversity (Zheng et al., 2022a), nutrient use efficiency (Zheng et al., 2022b), and stand productivity (Vangansbeke et al., 2015; Mao et al., 2017). Proper rotation and cutting intensity could maintain sustainable wood production in bamboo forests ( d’Oliveiraa et al., 2013). Franklin (2006) conducted harvesting experiments on wild Australian bamboo (*Bambusa arnhemica*) and found that harvesting had no effect on the number of new bamboos, however the diameter at breast height was significantly smaller than that of the control. In tropical secondary forests, the estimated return time is to 90% of that of the original biomass and not 100%. As the biomass becomes closer to the original level, more time is needed to achieve 100% complete recovery, and the gradual relationship with the recovery time can be shown by simulating the accumulation of biomass (Poorter et al., 2016). Moso bamboo is a typical uniaxial scattered bamboo species, which is connected by underground bamboo whips (Su et al., 2019). In the On-year, the mother bamboo uses physiological integration to transport nutrients to the daughter ramet on the whip through the underground rooting system, and many shoots are sprouted in the On-year (Song et al., 2016). However, the growth of many new trees had great nutrient consumption demands on the forest, which reduced the bamboo shoot yield in the next year (Su, 2012). In the Off-year, the germination of new bamboo shoots is reduced, and most of the photosynthate is used for the growth of underground whip roots (Li et al., 2019). The bamboo accumulated nutrients in the Off-year provide sufficient nutrients for the bamboo shoots in the next year, therefore the emergence of bamboo shoots alternated between On-year and Off-year (Su, 2012). As the structure and density of bamboo forests are constantly changing and they are poor self-fertilizers, short-term studies cannot fully elucidate the characteristics of stand restoration (Zheng et al., 2022b).

The characteristics of the stand and the variation in productivity after cutting is an important factor for people to explore strip clearcutting patterns for the sustainable management of Moso bamboo forest. To provide a theoretical basis for evaluating the quality restoration level of the strip clearcutting stand, plots that were strip cut with 8 m width (SC) and the reserved belts (RB) were selected as the research object and the traditional forest management (CK) as control. And the stand characteristics and biomass accumulation patters of each component of bamboo were monitored at the different sample plots for 5 years after the cutting. As Moso bamboo management is mainly concentrated in the On-year, the restoration dynamics of the bamboo stands were analyzed in the three On-years after cutting. It was hypothesized that (1) Cutting affects the new bamboo characteristics; (2) Cutting affects biomass allocation and productivity of the stand; and (3) with natural restoration in progress, the stand characteristics of SC could recover to the level of CK.

## Materials and methods

### Study site

**Figure 1:**
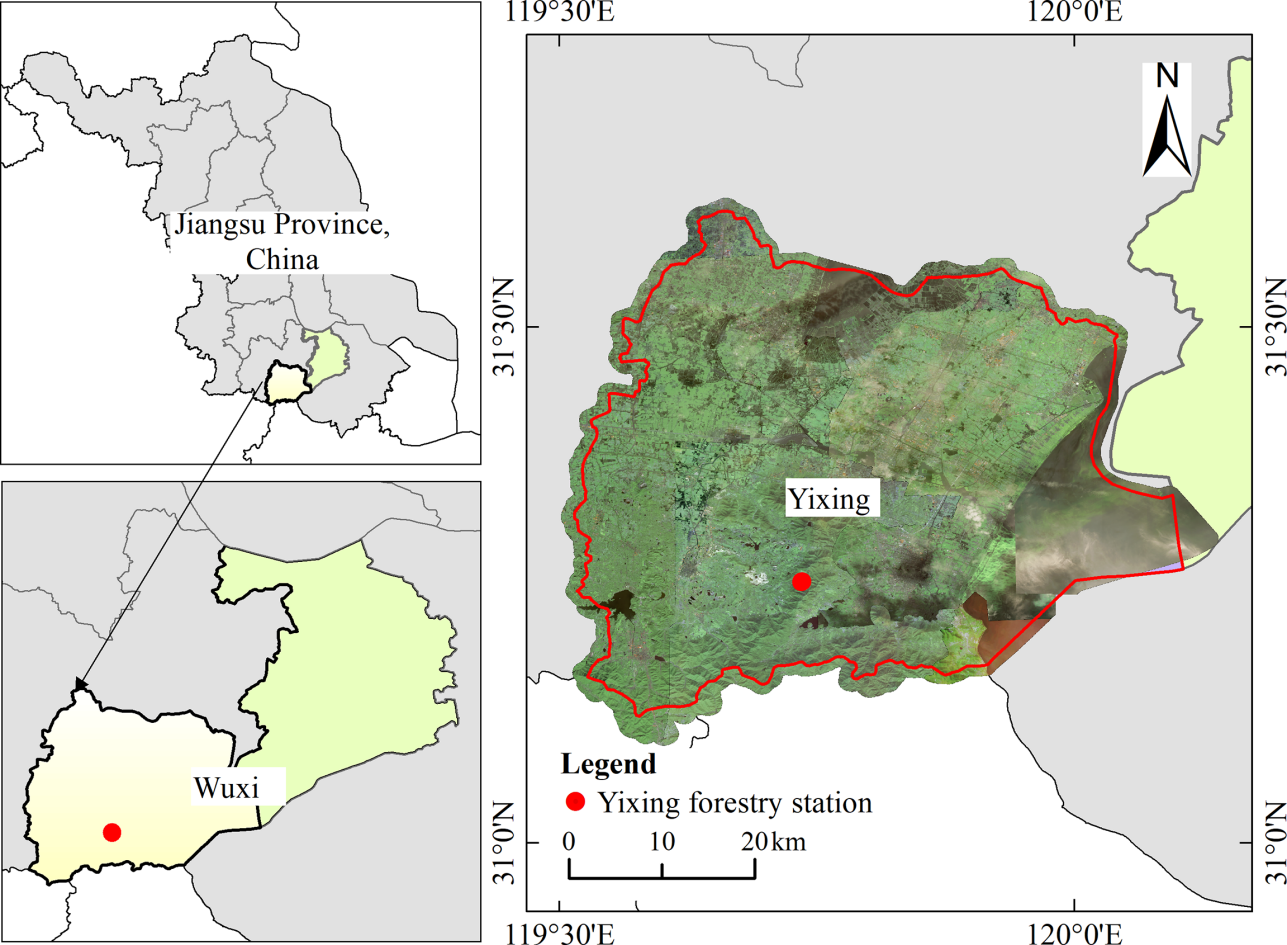
Location of the study area.

The study was undertaken on the Yixing forest farm (31°15′1″-31°15′12″ N, 119°44′2″-119°44′8″ E), located in southern Jiangsu Province, China (**Figure 1**). The experimental area is within the marine monsoon climate zone in the northern margin of the subtropics, with minimum and maximum temperatures of -4.5°C and 38.8°C, respectively. In addition, the average annual temperature is 16.5°C, and the average monthly temperature is 28.3°C in summer (data from Yixing Forest meteorological station, located 1 km from the field station). Rainfall occurs throughout the year, with an average annual rainfall of 129 days and average annual precipitation of 1229.9 mm, which is concentrated in spring and summer. The terrain is dominated by low hills. The predominant understory species include *Oxalis corniculata*, *Hedyotis chrysotricha*, *Paederia cruddasiana*, and *Salvia prionitis* (Zheng et al., 2021).

### Experimental design

Strip clearcutting refers to cutting all bamboo in the plot. On February 2017, pure Moso bamboo stands with the same management measures, slope, and the same stand structure were selected for the experiment. Three strip cut plots (SC) with a width of 8 m and a length of 20 m were set. Reserved plots (RB) with the same width were set on both sides of the SC. We calculated by weighing the biomass out of the cut and testing the nutrient content, 59384.76 ± 9864.53 kg of biomass out of the strip cut plots. Meanwhile, isolation trenches 50 cm wide and 50 cm deep were dug around the plots to eliminate the effect of physiological integration of moso bamboo through long-distance nutrient transport from adult culms to new bamboo through rhizomes (Su et al., 2019). Three 20 m×20 m traditional management plots were set as the control (CK). The CK plot followed the original management methods, including digging bamboo shoots and artificial selective harvest of old bamboo in on-year, and digging all bamboo shoots in off-year. The SC and RB were restored naturally, and no management measures were taken during the experiment. A continuous survey and sampling was conducted from the three treatment plots from 2017 to 2021. Based on the physiological characteristics of bamboo, age is usually denoted by “du”. A 1-year-old bamboo was labeled as I “du”. Thereafter, bamboo leaves changed every two years, and each time, the age of the bamboo leaves increased by I “du” (Tang et al., 2016). For example, 2− 3-year-old bamboos labeled II “du,” and III “du” represented a 4−year-old bamboo forest (Zheng et al., 2022b). According to the growth characteristics of the Moso bamboo forest, the monitoring period included three On-years (2017, 2019, 2021) and two Off-years (2018, 2020). The basic information of the plot before cutting is shown in **Table 1**.

**Table 1:** Basic information of sample plots.

Plot	Slope	Altitude	Density	Mean DBH	Mean height to crown base	Mean treeheight	Total nitrogen	Total phosphorus		Total potassium	
		(m)	(individual/hm^2^)	(cm)	(m)	(m)	(TN g/kg)	(TP g/kg)		(TK g/kg)	
SC1	5°	113	3250	8.68	4.10	12.91	1.42		0.26		9.39
SC2	6°	113	3861	9.13	4.60	13.75	1.33		0.27		9.26
SC3	6°	113	3187	8.49	5.09	13.21	1.48		0.26		9.31
RB1	6°	113	3452	8.83	4.76	12.85	1.59		0.26		9.27
RB2	6°	114	3815	8.88	4.06	13.22	1.21		0.26		9.25
RB3	5°	114	3687	9.38	5.07	13.53	1.43		0.26		9.40
CK1	6°	113	3657	9.06	4.23	13.33	1.28		0.26		9.18
CK2	6°	113	3756	8.77	4.43	13.63	1.45		0.26		9.28
CK3	5°	114	3948	8.90	4.40	13.21	1.47		0.25		9.56

### Sample plot survey

From 2017 to 2021, bamboo shoot dynamics, including the number of shoots (NS), and the number of shoots returned, were investigated every year after the bamboo shoots formed (May). Subsequent to branch drawing and leaf spreading (August), the newly emerging bamboos in the sample plot were investigated, including diameter at breast height (DBH), bamboo height (H), and height to crown base (HCB). We first cut a 13m moso bamboo as a reference, then marked it with paint every 1m, brought it to the sample plot, placed it next to the moso bamboo to be measured, and the same person visually measured HCB and H of the bamboo.

### Biomass survey

The average DBH, HCB, and H of Moso bamboo at different ages in different treatment plots were calculated. And the Moso bamboo that met the average characteristics of each age of the three treatment plots was selected as the corresponding standard bamboo. In November of each year, according to the statistical data, the standard bamboo was selected to investigate the biomass. To avoid the interference of standard bamboo harvesting on the plots, alternative methods were used to sample biomass of the plots according to the relevant information on the standard bamboo. Twelve standard bamboo were selected from each treatment totaling 36 bamboos. The entire plant is divided into branches, leaves, and culms. Samples were oven-dried at 65°C for 48 h to a constant weight and then weighed. The biomass of each organ of Moso bamboo at stand level was calculated according to Equation 1.


(1) $$W_{ij}=n_{i}w_{ij}$$

Where *i* is the age of bamboo, including I, II, and III “du”; *j* refers to different treatment plots, including SC, RB, and CK; *w_ij_* is the average dry weight of i-year-old standard bamboo branches, culms, and leaves in sample plot j; *n_i_* is the number of i-year-old moso bamboo plants; *W_ij_* is the total dry weight of branches, culms and leaves of i-year-old bamboo in plot j.

### Calculation of productivity

According to the biomass of each organ (branch, culm, and leaf) obtained from Moso Bamboo standard bamboo, the biomass of the different organs can be calculated. The specific calculation method is as Equation 2:


(2) $$\Delta w=\sum n_{i}(\bar{w_{i}}-\bar{w_{i-1}})$$

Where Δ*w* is the productivity of an organ of Moso bamboo, *i* is the age of bamboo, *n_i_* is the number of bamboo plants at the age of i, $\bar{w_{i}}$ is the biomass of an organ of standard bamboo at the age of i.

### Soil sampling

From 2017 to 2021, a soil drill was used to stratify soil along the median line (0–40 cm) in each experimental plot in November each year. Ten soil cores were taken from each plot. The mixed samples of soil composition were spread flat on clean paper and placed in a cool and ventilated place indoors to air dry. Stones and undecomposed organic matter were removed, ground, passed through a 2-mm sieve. They were thoroughly mixed, and a part of the sample was placed into a sealed bag for available nutrients determination. The remaining samples were passed through a 2-mm sieve, and were then further ground to pass through a 0.149-mm sieve for determination of soil organic matter (SOC), total nitrogen (TN), total phosphorus (TP), and total potassium content (TK).

### Chemical analysis

The soil organic carbon (SOC) and total nitrogen (TN) content were determined using an elemental analyzer (ECS 4024 CHNSO; Costech, Picarro, Italy). Total phosphorus (TP) content was determined following the molybdenum-antimony resistance colorimetric method (concentrated H2SO4-HClO4) using an automatic chemical analyzer (Smartchem 300; AMS, Italy). Total potassium (TK) content was determined using a flame photometer (M410; Sherwood, United Kingdom). Soil alkali-hydrolyzable nitrogen content (AN) was determined following the alkali-hydrolyzable diffusion method. A continuous flow analyzer (AA3, Seal, Germany) was used to determine the available soil phosphorus (AP) content. The content of available potassium (AK) was determined by flame atomic absorption spectrometry. And, pH was determined using an electrode pH meter (Sartorius PB-10, Germany).

### Statistical analysis

The differences in stand characteristics and productivity among different treatment plots were tested using a One-way analysis of variance (ANOVA). The assumptions of normality and homogeneous variance were examined using the Shapiro-Wilk test and Leven’s test, respectively. The means were separated by the least significant difference (LSD) test, and statistical significance was set at *p*<0.05. All statistical analyses were performed in R (version 3.6.2) (R Core Team, 2020), and the data were calculated using Excel 2016 (Britannica, 2019). A linear regression analysis was used to detect the relationship between the stand productivity and shoot dynamics. Lastly, Pearson correlation analysis was used to detect the relationship between stand productivity and soil nutrient content. All graphs were drawn using the ggplot2 package.

## Result

### New bamboo characteristics

**Figure 2:**
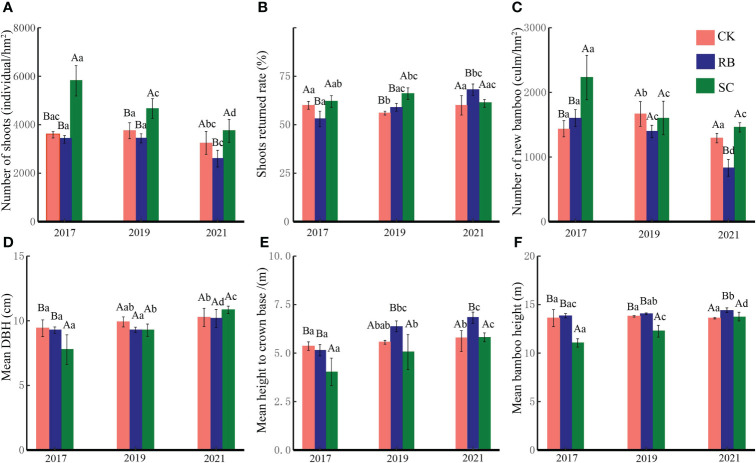
Shoot dynamics **(A)**, number of shoots; **(B)**, shoots returned rate; **(C)**, number of new bamboos), and new bamboo characteristics **(D)**, mean DBH; **(E)**, mean height to crown base; **F**, mean bamboo height) in different treatments plot. Different lowercase letters indicate that there are significant differences in the characteristics of new bamboo in the same treatment plot at different times (ANOVA and LSD test, *p* < 0.05). Different capital letters indicate that there are significant differences in the characteristics of new bamboo in different treatments at the same time (ANOVA and LSD test, *p* < 0.05). Error bars indicate standard deviation (n = 3).

In the first year after cutting, the number of shoots and new bamboo trees in SC were significantly higher than those in RB and CK (*p*<0.05) (**Figure 2**). With the natural recovery of the plots, the number of shoots and new bamboo trees gradually decreased in SC. After three years, the shoot returned rate in SC was significantly higher than that in RB and CK (*p*<0.05). After five years, the number of bamboo shoots and new bamboo trees in RB were significantly lower than those in SC and CK, and the shoot returned rate was significantly higher than that in SC and CK (*p*<0.05).

Cutting significantly reduced the average DBH, average height under branches and the average height of new bamboo trees (*p*<0.05). With the natural recovery of the plots, the average DBH, average height under branches, and the average height of new bamboo trees gradually increased in SC. After five years, there was no significant difference in the characteristics of new bamboo trees between SC and CK (*p*>0.05). The average height under branches and the average height of new bamboo trees in RB were significantly higher than those in SC and CK (*p*<0.05).

### Bamboo productivity

In the three treatment plots, the productivity of each organ of bamboo was the highest in culm, followed by branch, and then the lowest in leaf. Cutting did not reduce the productivity of Moso bamboo (**Table 2**). Over time, the productivity of branches and culms increased gradually, while the productivity of bamboo leaves gradually decreased in SC. However, the productivity of organs in RB gradually decreased.

**Table 2:** Productivity of different organs in different treatments plots.

Component	Time	CK	RB	SC
Branch (kg/hm^2^·a)	2017	2938.70 ± 546.68Aa	3058.83 ± 682.87Aa	2714.58 ± 907.78Aa
	2019	2903.55 ± 462.84Aa	2930.53 ± 469.91Aa	3854.34 ± 623.14Aa
	2021	2264.96 ± 794.71Aa	1260.91 ± 1146.60Ba	3865.56 ± 2494.84Aa
Culm (kg/hm^2^·a)	2017	19719.77 ± 3989.22Aa	15710.91 ± 4462.26Aba	13457.60 ± 3723.36Aa
	2019	21579.94 ± 864.57Aa	16943.97 ± 2583.54Aa	17077.46 ± 5240.94Aa
	2021	18426.76 ± 5860.49Aa	9976.97 ± 2734.19Ba	20109.00 ± 6025.35Aa
Leaf (kg/hm^2^·a)	2017	1430.44 ± 45.65ABa	1519.91 ± 108.30Aa	2041.10 ± 671.56Aa
	2019	1924.43 ± 697.52Ba	1650.31 ± 546.04Aa	2624.00 ± 490.83Aa
	2021	1211.75 ± 472.85ABa	900.93 ± 545.02Aa	1018.93 ± 234.44Aa

Values are the mean ± standard deviation (n = 3). Different lowercase letters indicate that there are significant differences in the component productivity of the new bamboo between different treatment plots at the same time (ANOVA and LSD test, p < 0.05); Different capital letters indicate that there are significant differences in the component productivity of new bamboo in different time at the same treatment plots (ANOVA and LSD test, p < 0.05).

**Figure 3:**
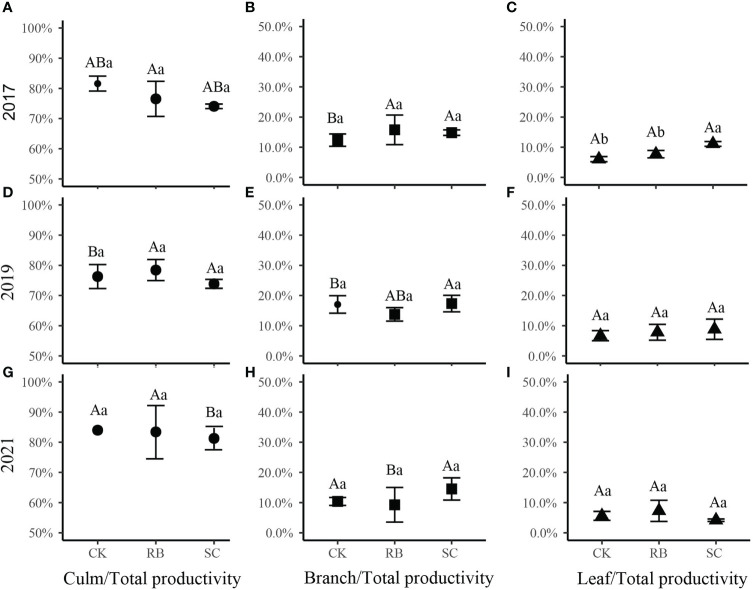
The ratio of each organ (**A, D, G**, culm; **B, E, H**, branch; **C, F, I**, leaf) productivity to aboveground productivity of Moso bamboo in different treatment plots. Different lowercase letters indicate that there are significant differences in the ratio between different treatment plots at the same time (ANOVA and LSD test, *p* < 0.05); Different capital letters indicate that there are significant differences in the ratio at different times at the same treatment plots (ANOVA and LSD test, *p* < 0.05). Error bars indicate standard deviation (n = 3).

Further analysis showed that in the first year after cutting, the percentage of leaf productivity to the total productivity in SC was higher than that in CK and RB (**Figure 3**, *p*<0.05). Five years after cutting, the percentage of branch productivity to total productivity in the RB decreased significantly, while, the percentage of culm productivity to total productivity in SC increased significantly (**Figure 3**, *p*<0.05).

### Stand characteristics after 5 years of cutting

Five years after cutting, the mean height to crown base, mean height, and mean DBH of Moso bamboo in the SC were still significantly lower than those in RB and CK (*p*<0.05). However, the density of bamboo in the different treatment plots following RB>SC>CK. Aboveground biomass storage in SC reached the level of CK (**Table 3**).

**Table 3:** Stand characteristics and aboveground biomass storage of bamboo under different treatments at five years after cutting.

Site	Mean Height to crown base	Mean Height	Mean DBH	Density	Aboveground biomass
	(m)	(m)	(cm)	(individual/hm^2^)	(kg/hm^2^)
SC	4.72 ± 0.19A	11.89 ± 0.06A	8.66 ± 0.31A	5583.33 ± 190.94A	73357.74 ± 7875.45AB
RB	5.99 ± 0.18B	13.91 ± 0.06B	9.19 ± 0.15B	8166.67 ± 815.70B	97568.93 ± 10763.71A
CK	5.58 ± 0.08C	13.66 ± 0.29B	9.86 ± 0.25C	4295.83 ± 260.21C	63728.99 ± 8469.32B

Values are the mean ± standard deviation (n = 3). Different capital letters indicate that there are significant differences in stand characteristics and aboveground biomass in different treatment plots (ANOVA and LSD test, p < 0.05).

## Discussion

### Difference in new bamboo characteristics

In the first year after cutting, the number of shoots and bamboo increased significantly. This may be due to the gibberellin content in whip buds, and soluble sugar, soluble protein, and the endogenous hormone of new bamboo increased after cutting (Wang, 2021). It was found that the increase in auxin accumulation in whip buds was the key to promoting shoot production (Wang, 2021). Soluble sugar and protein acted synergistically to promote germination and growth of shoots (Zeng et al., 2019). High gibberellin accumulation could reduce the number of bamboo shoots withdrawn (Chen et al., 2022). In addition, it was found that stronger light was beneficial to clone plants to produce more ramets (Li et al., 2018). The gap was formed in the bamboo forest after cutting, and the temperature heterogeneity of the stratum was good (Shen et al., 2020). With the recovery of the cut plots, the canopy structure returned to the control level, and the difference in temperature and humidity between the forest and the control plots decreased (Shen et al., 2020). The number of shoots and bamboo decreased significantly after the third On-year growth in RB. This was likely because the stand density was too high, and the nutrient competition among bamboo plants was fierce (Liu et al., 2016). The mean height to crown base and mean height of new bamboo gradually increased, mainly in high-density bamboo forests, by increasing the height growth to obtain nutrients. However, at the early stage of harvest recovery, the new bamboo height in SC reduction was owing to the content of non-structural carbohydrates in shoots and new bamboo was significantly lower than CK (Zeng, 2019). The carbon content in the shoot is not adequate for high growth (Song et al., 2016). In addition, trace elements in the soil could lead to the internode growth of Moso bamboo. A previous studies have found that zinc and boron deficiency can affect high growth of Moso bamboo (White, 2012).

**Figure 4:**
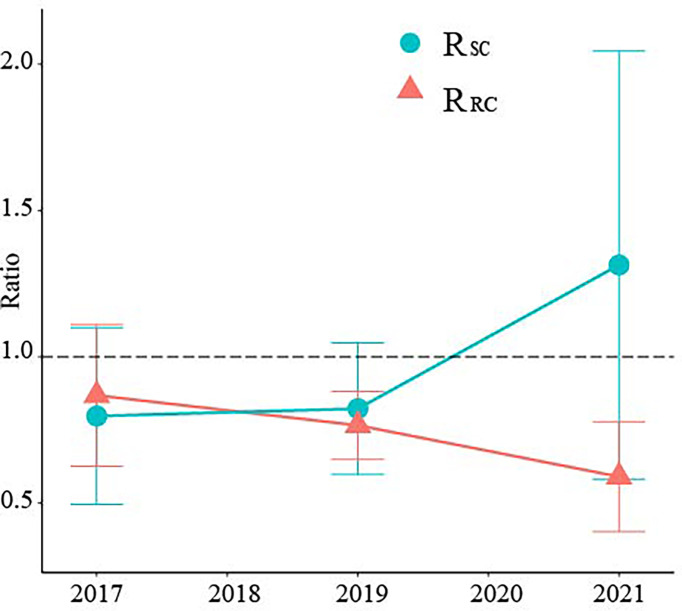
Ratio of aboveground productivity between different treatments in different years. RSC represents the aboveground productivity ratio of SC to CK; RRC represents the aboveground productivity ratio of RB to CK. Error bars indicate standard deviation (n = 3).

Reducing the height of the crown base and allocating more nutrients to leaves are morphological plasticity strategies for new bamboo to adapt to strip harvesting (Zheng et al., 2021). Allocating more biomass to bamboo branches and leaves could speed up bamboo forest recovery, as higher LAI may lead to higher light capture and total biomass increase (Prado-Junior et al., 2016). In the first year after cutting, the productivity ratio of RB and SC to CK was less than 1. Cutting reduced the productivity of RB and SC (**Figure 4**). Therefore, this verifies our hypothesis. The reduction in bamboo density in SC reduced the stand productivity (Liu, 2009). And nutrient supply from the RB to SC through physiological integration may limit the growth and development of standing bamboo in RB (Li et al., 2000). With increasing time, the productivity of SC increased, while that of RB decreased. At 5 years after cutting, the productivity of SC was higher than that of CK, while that of RB was lower than that of CK. This was owing to the high number of old bamboo (Su, 2012) and high stand density (Liu et al., 2016). Density regulation is an important measure to prevent productivity degradation of bamboo forests (Liu et al., 2016).

### Correlation between shoots dynamics and productivity

**Figure 5:**
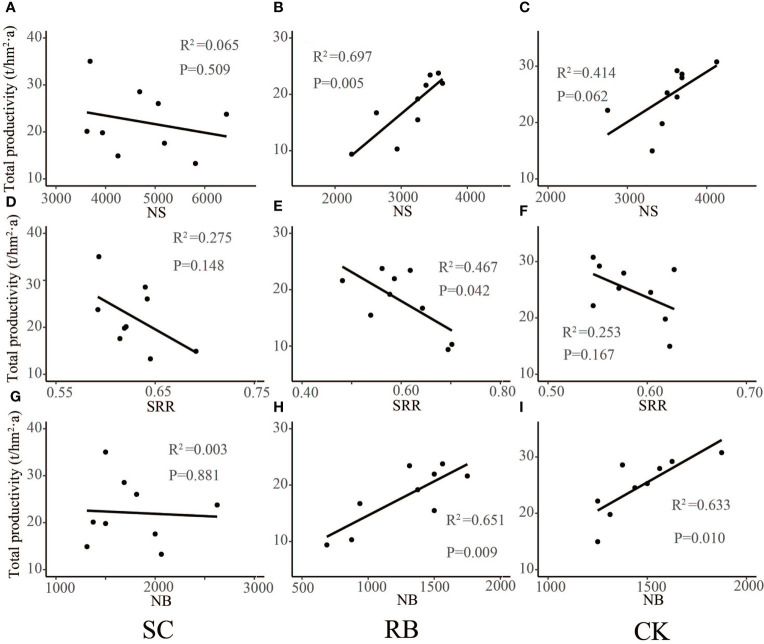
Liner regression analysis between shoots dynamics **(A–C)**, number of shoots; **(D–F)**, shoots returned rate; **(G–I)**, number of new bamboos) and productivity in different treatment plots.

The physiological and metabolic levels of Moso bamboo at different ages were very different (Umemura and Takenaka, 2014). It was found that the biomass of I “du” bamboo was significantly lower than that of II “du” and III “du”, and the biomass difference between II “du” and III “du” was small. This meant that the growth rate of dry matter accumulation of Moso bamboo decreased significantly after the growth of II “du”, and the biomass accumulation of each organ of Moso bamboo gradually slowed down when the growth of III “du” grew to IV “du” (Su, 2012). We found that in CK, productivity positively correlated with the number of bamboo trees (**Figure 5**, *p*<0.05). In SC, shoot dynamics did not significantly relate to productivity (**Figure 5**). The amount of standing bamboo of at II “du” and III “du” in SC was greater than that in CK, and the number of new bamboos did not decrease (**Figure 2**). This meant that the growth of new bamboo was less than the contribution of productive, and the main contribution of productivity is the growth and development of the existing bamboo. In contrast, in RB, the number of shoots and the amount of new bamboo positively correlated with productivity. In addition, the shoot returned rate significantly negatively correlated with productivity (**Figure 5**, *p*<0.05). This means that the growth of new bamboo is the main factor in the change of productivity in RB.

### Correlation between soil nutrients and productivity

The mineral elements needed for the growth and development of Moso bamboo include N, K, P (Embaye et al., 2005), Si, Ca (Ma and Takahashi, 2002), Zn, and B (White, 2012). In addition to N, P, and K, the contents of other elements in the soil could meet the growth of Moso bamboo (Su, 2012). Nitrogen is crucial for the yield from bamboo forests as the demand and absorption of N are highest during each growth period (Su et al., 2019). Phosphorus is an essential macronutrient for higher plants and is typically a highly mobile and frequently translocated element (White, 2012). Potassium accumulates in meristem and larval tissues and is absorbed by the roots of higher plants (White, 2012). Potassium has a high reabsorption efficiency in plants (Umemura and Takenaka, 2014).

**Figure 6:**
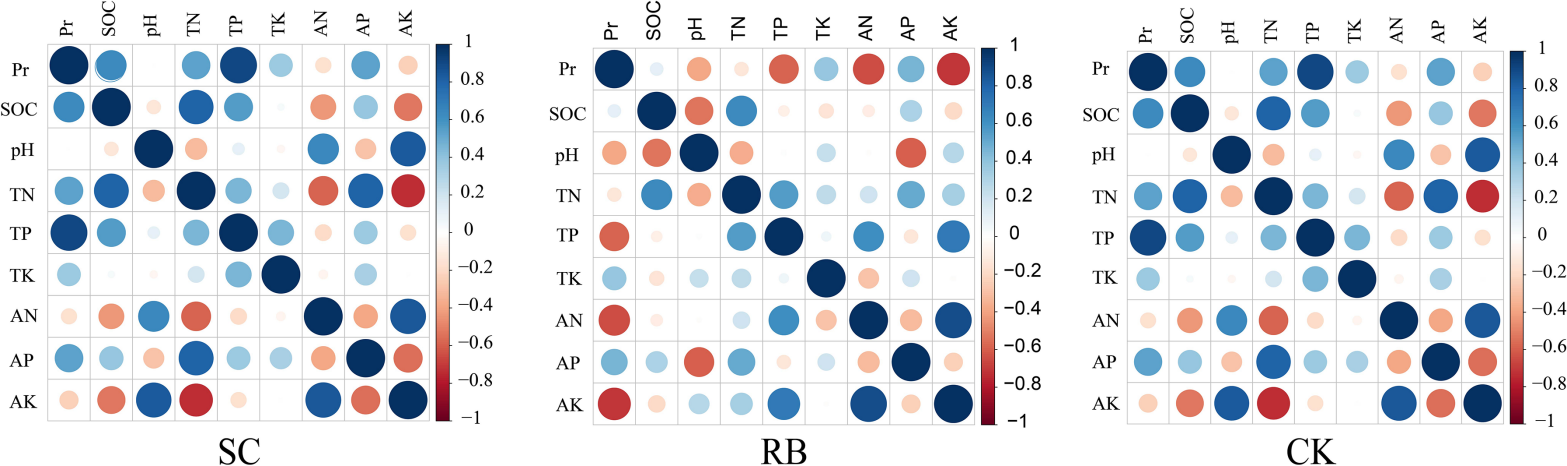
Correlation between productivity and soil nutrients in strip cut with 8m width (SC) and its reserved belts (RB), and the traditional management forest (CK). Pr is the bamboo productivity.

The study found that there are obvious differences in nutrient element storage of different components (Embaye et al., 2005). As a photosynthetic organ, the contents of nitrogen, phosphorus, and potassium in bamboo leaves were significantly higher than that in other organs (Su, 2012). The storage of N and K in culm and branch is higher than that of P. Furthermore, the nitrogen content in the organs of I “du” and II “du” bamboo were slightly higher than that of III “du” bamboo, while the phosphorus content of II “du” and III “du” bamboo was higher than I “du” bamboo (Liu, 2009). According to the above analysis, the productivity in SC and CK was mainly contributed by the II “du” and III “du” Moso bamboo, and in RB was mainly contributed by the growth of new bamboo. This means that more N and P are needed for bamboo growth in SC and CK. Our correlation analysis showed that the soil contents of total N, total P, and available P positively correlated with productivity in SC and CK (**Figure 6**). As a cofactor of various enzymes, the K content of I “du” bamboo was significantly higher than that of II “du” and III “du” bamboo (Umemura and Takenaka, 2014). In our study, the productivity of RB negatively correlated with the contents of total P, available N, and available K in soil (**Figure 6**). This is inconsistent with the demand for bamboo management. A fertilization experiment of Moso bamboo revealed that nutrient addition could increase aboveground biomass (Ni et al., 2021). In contrast, we believe that the increase in available potassium content in the soil was due to the excessive density of standing bamboo, and rainfall brings more K^+^ to the soil through leaching (Umemura and Takenaka, 2014). Meanwhile, it is believed that the bamboo forest density restricts productivity and decouples stand productivity from soil nutrient change. Thus, it is suggested that the productivity of RB is not limited by nutrient deficiency and can be managed.

### Implication for bamboo forest management

The main considerations for the feasibility of strip cutting in the bamboo forest are the nutrient supply capacity of the mother bamboo and the restoration level of strip cut plots (Zheng et al., 2022a). Strip cutting experiments on different bamboo species have been carried out in Jiangsu, Anhui, Yunnan, Sichuan, and Fujian (Zeng, 2019; Zhan, 2019; Wu, 2020; Wang, 2021), covering most of the regions of bamboo producing areas in China (Administration, 2019). Studies on strip cutting of Moso bamboo have concluded relatively consistent results regarding the setting of the cutting width and the recovery period (Zeng, 2019; Zhan, 2019; Wu, 2020). Thus, we believe that the strip cutting method is suitable for the management of moso bamboo forests nationwide. It was found that increasing the quantity and quality of bamboo in the first year after cutting is an important measure to restore productivity. It is suggested to fertilize the cutting area in advance before cutting to ensure the high-quality growth of bamboo. However, in different regions, soil nutrients, topographic environment, and climate factors are different, and the amount and types of nutrients added are different, which needs further research.

## Conclusion

The number of new bamboo trees increased, however the productivity of the stand decreased in the early stage of strip cutting restoration. After 5 years of natural restoration, the new bamboo characteristics, bamboo density, and productivity of SC could be restored to the CK level; however the stand productivity of RB decreased. When considering bamboo forest management, it can be said that 6-year is the optimal restoration period for the strip cut plots, and the bamboo forests could be harvested by strip cutting after 6 years of restoration. In addition, it is suggested that before strip cutting, advanced fertilization could be utilized to increase the nutrient content in the soil, promote high-quality growth of bamboo, and improve the restoration level of plots. After 2 years of restoration, all bamboo shoots within the reserve belt plots should be harvested to improve economic benefits and nutrient utilization efficiency.

## Data availability statement

The raw data supporting the conclusions of this article will be made available by the authors, without undue reservation.

## Author contributions

SF and FG designed this study and improved the English language and grammatical editing. YZ wrote the first draft of manuscript, and performed the data analysis. YZ and XiZ did the field works. XuZ gave guidance and methodological advice. All the coauthors contributed to the discussion, revision and improvement of the manuscript.

## Funding

This research was supported by the Basic Scientific Research Funding of International Center For Bamboo and Rattan (1632022004).

## Conflict of interest

The authors declare that the research was conducted in the absence of any commercial or financial relationships that could be construed as a potential conflict of interest.

## Publisher’s note

All claims expressed in this article are solely those of the authors and do not necessarily represent those of their affiliated organizations, or those of the publisher, the editors and the reviewers. Any product that may be evaluated in this article, or claim that may be made by its manufacturer, is not guaranteed or endorsed by the publisher.

## References

[B1] Administration, N.F.A.G (2019). China Forest resources report (Beijng: China Forest Publishing House).

[B2] Britannica (2019) The editors of encyclopaedia. Microsoft excel. encyclopedia Britannica. Available at: https://www.britannica.com/technology/Microsoft-Excel.

[B3] Chen M. Guo L. Ramakrishnan M. Fei Z. Vinod K. K. Ding Y.. (2022). Rapid growth of moso bamboo (*Phyllostachys edulis*): Cellular roadmaps, transcriptome dynamics, and environmental factors. Plant Cell, 34 (10), 3577–3610. doi: 10.1093/plcell/koac193 PMC9516176 35766883

[B4] d’Oliveiraa M. V. N. Guarinoa E. d. S. Oliveiraa L. C. Ribasa L. A. Acuñab M. H. A. (2013). Can forest management be sustainable in a bamboo dominated forest? a 12-year study of forest dynamics in western Amazon. For. Ecol. Manage. 310, 672–679. doi: 10.1016/j.foreco.2013.09.008

[B5] Embaye K. Weih M. Ledin S. Christersson L. (2005). Biomass and nutrient distribution in a highland bamboo forest in southwest Ethiopia: Implications for management. For. Ecol. Manage. 204, 159–169. doi: 10.1016/j.foreco.2004.07.074

[B6] Fan S. Liu G. Su W. Cai C. Guan F. (2018). ). advances in research of bamboo forest cultivation. For. Res. 31, 137–144. doi: 10.13275/j.cnki.lykxyj.2018.01.01

[B7] Franklin D. C. (2006). Wild bamboo stands fail to compensate for a heavy 1-year harvest of culm shoots. For. Ecol. Manage. 237, 115–118. doi: 10.1016/j.foreco.2006.09.036

[B8] Jiang Z. H. (2007). Bamboo and rattan in the world (Beijng: China Forest Publishing House).

[B9] Li Y. Chen J.-S. Xue G. Peng Y. Song H.-X. (2018). Effect of clonal integration on nitrogen cycling in rhizosphere of rhizomatous clonal plant, *Phyllostachys bissetii*, under heterogeneous light. Sci. Total Environ. 628-629, 594–602. doi: 10.1016/j.scitotenv.2018.02.002 29454200

[B10] Li L. Li N. Lu D. Chen Y. (2019). Mapping moso bamboo forest and its on-year and off-year distribution in a subtropical region using time-series sentinel-2 and landsat 8 data. Remote Sens. Environ. 231, 111265. doi: 10.1016/j.rse.2019.111265

[B11] Liu G. (2009). Study on the mechanism of maintaining long-term productivity of bamboo forest. Chin. Acad. Forest.

[B12] Liu G. Shi P. Xu Q. Dong X. Wang F. Wang G. G.. (2016). Does the size-density relationship developed for bamboo species conform to the self-thinning rule? For. Ecol. Manage. 361, 339–345. doi: 10.1016/j.foreco.2015.11.030

[B13] Li R. Werger M. J. A. Kroon H. D. During H. J. Zhong Z. C. (2000). Interactions between shoot age structure, nutrient availability and physiological integration in the giant bamboo phyllostachys pubescens. Plant Biol. 2, 437–446. doi: 10.1055/s-2000-5962

[B14] Li P. Zhou G. Du H. Lu D. Mo L. Xu X.. (2015). Current and potential carbon stocks in moso bamboo forests in China. J. Environ. Manage. 156, 89–96. doi: 10.1016/j.jenvman.2015.03.030 25836664

[B15] Mao F. Zhou G. Li P. Du H. Xu X. Shi Y.. (2017). Optimizing selective cutting strategies for maximum carbon stocks and yield of moso bamboo forest using BIOME-BGC model. J. Environ. Manage. 191, 126–135. doi: 10.1016/j.jenvman.2017.01.016 28092748

[B16] Ma J. F. Takahashi E. (2002). Soil, fertilizer, and plant silicon research in Japan (Amsterdam: Elsevier Science).

[B17] Ni H. Su W. Fan S. Chu H. (2021). Effects of intensive management practices on rhizosphere soil properties, root growth, and nutrient uptake in moso bamboo plantations in subtropical China. For. Ecol. Manage. 493, 119083. doi: 10.1016/j.foreco.2021.119083

[B18] Poorter L. Bongers F. Aide T.M. Almeyda Zambrano A. M. Balvanera P. Becknell J. M. (2016). Biomass resilience of Neotropical secondary forests. Nature 530, 211–214. doi: 10.1038/nature16512 26840632

[B19] Prado-Junior J. A. Schiavini I. Vale V. S. Arantes C. S. van der Sande M. T. Lohbeck M.. (2016). Conservative species drive biomass productivity in tropical dry forests. J. Ecol. 104, 817–827. doi: 10.1111/1365-2745.12543

[B20] R Core Team (2020). R: A language and environment for statistical computing (Vienna, Austria: R Foundation for Statistical Computing). Available at: http://www.R-project.org/.

[B21] Shen J. Fan S. Liu G. Chen B. Wu C. Cao B. (2020). Spatiotemporal distribution characteristics of temperature on the surface layer of cutting gap of *Phyllostachys edulis* forest. Chin. J. Ecol. 39, 3549–3557. doi: 10.13292/j.1000-4890.202011.024

[B22] Song X. Peng C. Zhou G. Gu H. Quan L. Chao Z. (2016). Dynamic allocation and transfer of non-structural carbohydrates, a possible mechanism for the explosive growth of moso bamboo (*Phyllostachys heterocycla*). Sci. Rep. 6, 25908. doi: 10.1038/SREP25908 27181522 PMC4867622

[B23] Su W. (2012). Fertilization theory and practice for phyllostachys edulis stand based on growth and nutrient accumulation rules. Chin. Acad. Forest.

[B24] Su W. Fan S. Zhao J. Cai C. (2019). Effects of various fertilization placements on the fate of urea-^15^N in moso bamboo forests. For. Ecol. Manage. 453, 117632. doi: 10.1016/j.foreco.2019.117632

[B25] Tang X. Qi L. Fan S. Guan F. Zhang H. (2016). Soil respiration and net ecosystem production in relation to intensive management in moso bamboo forests. Catena 137, 219–228. doi: 10.1016/j.catena.2015.09.008

[B26] Umemura M. Takenaka C. (2014). ). retranslocation and localization of nutrient elements in various organs of moso bamboo (*Phyllostachys pubescens*). Sci. Total Environ. 493, 845–853. doi: 10.1016/j.scitotenv.2014.06.078 25000580

[B27] Vangansbeke P. De Schrijver A. De Frenne P. Verstraeten A. Gorissen L. Verheyen K. (2015). Strong negative impacts of whole tree harvesting in pine stands on poor, sandy soils: A long-term nutrient budget modelling approach. For. Ecol. Manage. 356, 101–111. doi: 10.1016/j.foreco.2015.07.028

[B28] Wang S. (2021). Study on response characteristics of underground whip root system and ground growth of phyllostachys edulis forests under different strip cutting Ph.D (Beijing, China: Chinese Academy of Forestry).

[B29] White P. J. (2012). long-distance transport in the xylem and phloem (Invergowrie, UK: Elsevier).

[B30] Wu C. M. (2020). Early response of soil microbial community structure after strip cutting in moso bamboo forest in central fujian (Beijing, China: Chinese Academy of Forestry).

[B31] Zeng X. L. (2019). Recovery characteristics and influencing factors of moso bamboo forests under different strip clearcutting in south anhui province. Ph.D (Beijing, China: Chinese Academy of Forestry).

[B32] Zeng X. L. Su W. H. Fan S. H. Jin Y. (2019). Qualitative recovery characteristics of moso bamboo forests under strip clearcutting. Acta Botanica Boreali Occidentalia Sin. 39, 917–924. doi: 10.7606/j.issn.1000-4025.2019.05.0917

[B33] Zhan M. C. (2019). Characteristic research on rapid regeneration of strip harvesting bamboo (Phyllostachys edulis) forest in jiangsu province (Beijing, China: Chinese Academy of Forestry).

[B34] Zheng Y. Fan S. Guan F. Xia W. Wang S. Xiao X. (2022a). Strip clearcutting drives vegetation diversity and composition in the moso bamboo forests. For. Sci. 68, 27–36. doi: 10.1093/forsci/fxab044

[B35] Zheng Y. Guan F. Fan S. Yan X. Huang L. (2022b). Dynamics of leaf-litter biomass, nutrient resorption efficiency and decomposition in a moso bamboo forest after strip clearcutting. Front. Plant Sci. 12. doi: 10.3389/fpls.2021.799424 PMC8829046 35154189

[B36] Zheng Y. Guan F. Fan S. Zhou Y. Jing X. (2021). Functional trait responses to strip clearcutting in a moso bamboo forest. Forests 6, 793. doi: 10.3390/f12060793

